# Determining the effect of different cooking methods on the nutritional composition of salmon (*Salmo salar*) and chilean jack mackerel (*Trachurus murphyi*) fillets

**DOI:** 10.1371/journal.pone.0180993

**Published:** 2017-07-07

**Authors:** José M. Bastías, Pamela Balladares, Sergio Acuña, Roberto Quevedo, Ociel Muñoz

**Affiliations:** 1 Department of Food Engineering, University of Bío-Bío, Chillán, Chile; 2 Department of Aquaculture and Agri-Food Resources, University of Los Lagos, Osorno, Chile; 3 Institute of Food Science and Technology, University Austral of Chile, Valdivia, Chile; The Pennsylvania State University, UNITED STATES

## Abstract

The effect of four cooking methods was evaluated for proximate composition, fatty acid, calcium, iron, and zinc content in salmon and Chilean jack mackerel. The moisture content of steamed salmon decreased (64.94%) compared to the control (68.05%); a significant decrease was observed in Chilean jack mackerel in all the treatments when compared to the control (75.37%). Protein content in both salmon and Chilean jack mackerel significantly increased under the different treatments while the most significant decrease in lipids was found in oven cooking and canning for salmon and microwaving for Chilean jack mackerel. Ash concentration in both salmon and Chilean jack mackerel did not reveal any significant differences. Iron and calcium content only had significant changes in steaming while zinc did not undergo any significant changes in the different treatments. Finally, no drastic changes were observed in the fatty acid profile in both salmon and Chilean jack mackerel.

## Introduction

Fish is one of the most complete foods and provides nutrient quality and quantity; an average 100 g portion provides more than 50% of the recommended daily protein intake, between 10% and 20% of minerals, variable quantities of water-soluble vitamins, and an important percentage of liposoluble vitamins A, D, and E [1]. A number of health benefits are attributable to fish; it is recommended that fish be compulsory in the diet. Different studies have demonstrated that there is an inverse relationship between fish consumption and the incidence of heart and immunological diseases in which fatty acids play a relevant role [2]. Fish fat is characterized by its high polyunsaturated fatty acid (PUFA) content, especially eicosapentaenoic acid (EPA) 20:5 n-3 and docosahexaenoic acid (DHA) 22:6 n-3 of the omega-3 series that are extremely important as cellular membrane components [3]. It has also been reported that these fatty acids markedly reduce plasma triglyceride levels, fulfill different physiological functions, such as reducing plasma low-density lipoprotein cholesterol levels, and exhibit antithrombotic, anti-inflammatory, antiarrhythmic, and vasodilating properties [3–7].

The nutritional value of fish can be established by quantifying the protein and fat (rich in PUFAs) content, as well as determining the concentration of minerals, such as calcium (Ca), iron (Fe), and zinc (Zn) [8]. On the other hand, information about nutritional value and chemical composition comes from international tables that are often constructed with data obtained from raw foods. The PUFA content in raw fish tissue cannot provide explicit information about the nutritional value of these species after cooking [9]. Although fish is sometimes consumed raw in some preparations, such as sushi and ceviche, it usually undergoes a cooking process before being consumed: these cooking processes can lead to important changes in composition [5]. Different fish species and cooking method used can be determining factors for essential fatty acid content in the consumed products [10]. Some of the principal changes that occur during processing and final preparation of cooked food are due to oxidation. The PUFAs, such as EPA and DHA, are considered to be especially susceptible to oxidation during heating and other culinary treatments; this reaction is catalyzed by heat, light, trace metals, or enzymes, and it consists in generating free radicals [4]. It can also provoke protein denaturation and mineral solubilization [10–11]. The objective of the present study was to determine the effect of cooking processes on the proximate composition, fatty acid profile, and levels of Ca, Fe, and Zn in two of the most consumed species in Chile, such as: salmon (*Salmo salar*) and Chilean jack mackerel (*Trachurus murphyi)*.

## Experimental

### Raw materials

Salmon (*Salmo salar*) and Chilean jack mackerel (*Trachurus trachurus*) bulk fresh fillets, similar sizes (35–45 cm) and weights (0.48–0.56 kg), were purchased at the fishing terminal of the city of Chillán, Chile; they were immediately stored in containers with ice and transported to the food analysis laboratory of the University of Bío-Bío for processing and analysis.

### Cooking methods

Each cooking process was performed three times and in triplicate.

An electronic food steamer (OSTER 5711) was used for steaming; cooking time was 25 min and center temperature was 83.5 ± 2°C.

Oven cooking was carried out in an electric oven (THOMAS TH-25N) preheated at 250°C. Once this temperature was reached, fillets were cooked for 20 min, then turned over and cooked for another 5 min; center temperature was 76.2 ± 0.5°C.

Microwaving (THOMAS TH-20DM microwave) was carried out at 1500 W for 8 min; the fillet was then turned over and the cooking process continued for another 4 min. Mean center temperature after cooking was 86.3 ± 1°C.

Canned fillets were put into half pound tin cans that were then filled with a 2% saline solution; cans were placed in an exhauster to eliminate oxygen and were finally sealed and placed in the autoclave. Total sterilization time was 45 min with F_0_ 6 min and center temperature reached 121°C [12].

### Analysis

#### Proximate analysis

Proximate analysis was performed by methods described in AOAC [13]; 1 g of the ground samples was oven-dried for 5 h at 105°C and the moisture content was determined weighing the sample, which was expressed as percentage wet basis (wb) (Memmert 750). Protein content (Nx6.25) was established by the Kjeldahl method; for ash content, samples were incinerated in a muffle furnace (Nabertherm, L3/P) at 550°C until ashes were white. Total fat was determined using the methodology proposed by Bligh and Dyer [14].

#### Fatty acid profile

The fatty acid composition of the fish fillets, was determined by gas chromatography. Fat (20–40 mg) was saponified with 1 mL NaOH 0.5M in methanol, heated at reflux for 10 min, and then esterified using the methodology described by Hartman and Lago [15]. The methyl esters of fatty acids were analyzed with a Shimadzu GC-2010 gas chromatograph with an FID detector using a Rt 2560 capillary column (100 m, 0.25 mm ID, 0.2 μm df). Both injector and detector temperature was 250°C. Separation was carried out by a temperature ramp program with an initial temperature of 140°C that was increased by 10°C min^-1^ to 200°C and held for 20 min; there was a further increase of 2°C min^-1^ until reaching the final temperature of 240°C that was held for 10 min. Helium was the carrier gas and injection was performed in “Split” mode (1:100). Fatty acid methyl esters were identified by comparing them with retention times of a mixture of saturated fatty acid standards (C4-C24) (1000 μg/ml in hexane, analytical standard, 49453-U Supelco) and PUFAs (C4-C24) (wt% varied, analytical standard, 18919–1 AMP Supelco).

#### Mineral quantification

Samples were calcined and ashes were dissolved in 1 mL concentrated HCl; samples were then filtered and volume was adjusted to 25 mL deionized water (18.2 MΩ.cm) in accordance with the methodology described by AOAC [16]. The ash acid solution was analyzed for Ca, Fe, and Zn content. Mineral analysis was performed by flame atomic absorption spectrometry (FAAS) (VARIAN, model SPECTRA A-55).

### Statistical analysis

The statistical analysis was based on the one-way analysis of variance (ANOVA) of the mean values to test the statistically significant differences. All statistical tests were performed with the Statgraphic Centurion 16.1 software package. Mean values and standard deviations were used.

## Results and discussion

### Proximate composition

Changes in sample moisture, protein, fat, and ash contents after the cooking processes are shown in Table 1. Proximate composition of raw salmon and Chilean jack mackerel fillets differed with results revealed by Quitral et al. [17] for salmon fillets, and Toppe et al. [8] for Chilean jack mackerel. The chemical composition of fish muscle is very often different within the same species; this is influenced by many variables such as age, physiological state, and time and area of capture [18].

**Table 1:** Comparison of proximate composition (on a wet basis) of canned, oven-baked, microwaved, and steamed salmon and Chilean jack mackerel expressed as g/100g.

	Moisture		Proteins		Lipids		Ashes	
Treatment	Salmon	Chilean jack mackerel	Salmon	Chilean jack mackerel	Salmon	Chilean jack mackerel	Salmon	Chilean jack mackerel
Raw	68,05 ±2,24^ab^	75,37± 0,91^a^	18,66± 0,38^d^	16,58± 0,88^c^	11,98± 0,84^a^	6,37±0,21^a^	1,23± 0,17^a^	0,90± 0,09^ab^
Oven cooking	67,75± 0,60^abc^	68,35 ±1,56^b^	22,09 ±0,14^a^	25,36± 0,76^ab^	8,19 ±0,23^b^	5,15± 0,90^b^	1,29 ±0,75^a^	1,02 ±0,04^a^
Canning	70,17 ±0,54^a^	68,86± 0,77^b^	20,57± 0,23^b^	25,45± 0,05^ab^	7,32± 0,12^b^	4,65 ±0,66^b^	1,27± 0,03^a^	0,98± 0,15^ab^
Microwaving	65,48± 2,44^ab^	69,87±1,78^b^	19,89± 0,18^c^	24,48 ±1,14^b^	10,50± 1,26^a^	3,26 ±0,74^c^	1,32± 0,15^a^	0,98 ±0,05^ab^
Steaming	64,94 ±1,15^c^	65,72± 2,18^c^	21,95 ±0,31^a^	26,43± 0,26^a^	11,05± 1,28^a^	5,75± 0,52^ab^	1,39 ±0,89^a^	0,89± 0,02^b^

Different letters in the same column indicate significant differences between treatments within each species, p < 0.05.

Salmon fillet moisture content remained stable after cooking and exhibited no significant differences with the raw sample (Table 1), with the exception of steamed salmon fillets (64.94 g/100g). The moisture content of all the Chilean jack mackerel fillets was different from the raw sample and decreased after cooking. In addition, the moisture content of the steamed Chilean jack mackerel sample was significantly lower than the rest of the samples from the other cooking processes, and its behavior was similar to the salmon fillets.

This variation can be due to the difference in water retention capacity (WRC) of the salmon and Chilean jack mackerel muscle, which is caused by the capture/slaughter method used for both species. The fluctuation in WRC is an indicator of the changes in the myofibrillar protein structure; therefore, once subjected to the same culinary process, Chilean jack mackerel and salmon samples exhibit similar moisture contents (Table 1).

Protein content increased after cooking in all the evaluated methods and for both species under study. Lipid content decreased in the salmon fillets subjected to oven cooking and canning whereas lipid content in Chilean jack mackerel fillets also decreased with microwaving. Ash content showed no significant changes after cooking in both species under study.

The decrease in moisture content after heat treatments is caused by partial water loss through evaporation [5, 19]. Decreased moisture content has been described as the most important change causing significant protein increase in cooked fish fillets. Ersoy and Özeren [19] pointed out that the increase in protein, fat, and ash contents in fish after different cooking methods could be explained by water reduction, indicating an inverse relationship between water content and other nutritional components.

Lipid content significantly decreased after being subjected to the different cooking methods; this decrease is associated with cooking time and temperature, that is, lipid oxidation will be higher when cooking time is longer and temperature is higher [20]. The heat source must also be considered, especially for microwaving where microwaves can interact with unsaturations (double bonds) that are typical of lipids [5]. The first developments in the field of nutrition revealed that certain substances, such as total lipids and fatty acids, important for the proper functioning of the human body, are lost during cooking processes [21]. The heat treatment causes muscle protein denaturalization, which is the primary mechanism leading to moisture loss, and released water drags some lipids and protein solids (collagen, muscle tissue fragments, and sarcoplasmic proteins) [11]. Cooking stimulates water loss in food, which in turn increases its lipid content in most cases, and only a little fat is lost in fattier fish; this depends on the method and cooking time [5].

For ash content, no significant differences were observed in either salmon or Chilean jack mackerel compared to the control samples; these results are consistent with those found by Hosseini et al. [22] for Caspian white fish (*Rutilus frisii kutum*). Some authors [1, 19] have suggested a possible increase after cooking while others [23] have indicated a possible decrease in ash content because of losses related to lixiviation of these components that are lost with water and diffused when the muscle comes in contact with steam.

#### Mineral content

The concentrations of Ca, Fe, and Zn in raw salmon and Chilean jack mackerel are shown in Table 2. Calcium was the mineral with the highest concentration, 2.51mg/100g and 20.03 mg/100g for salmon and Chilean jack mackerel, respectively. Iron concentrations were 1.94 mg/100g and 2.81 mg/100g for salmon and Chilean jack mackerel, respectively, whereas Zn had the lowest concentrations, 0.46 mg/100g for salmon and 0.79 mg/100g for Chilean jack mackerel. The inedible parts (bones) were removed from the salmon and Chilean jack mackerel fillets when the analyzed samples were prepared. It is noteworthy that minerals are highly variable within the same species because their content depends on the season of the year, feeding habits, maturity stages, reproductive cycle, size, and biotic and abiotic factors among others [1, 24].

**Table 2:** Iron, calcium, and zinc content (expressed on a wet basis) in oven-baked, canned, microwaved, and steamed salmon and Chilean jack mackerel (mg/100g).

	Iron		Calcium		Zinc	
Treatment	Salmon	Chilean jack mackerel	Salmon	Chilean jack mackerel	Salmon	Chilean jack mackerel
Raw	1,94±0,43^a^	2,81±0,36^a^	2,51± 0,99^b^	20,03±4,66^a^	0,46±0,11^a^	0,79±0,18^a^
Oven cooking	0,58±0,13^c^	2,86±0,24^a^	2,37±0,62^b^	12,44±2,54^b^	0,45±0,08^a^	0,88±0,25^a^
Canning	0,61±0,08^c^	2,14±0,53^b^	3,98±0,38^a^	11,62±2,18^b^	0,45±0,07^a^	0,78±0,25^a^
Microwaving	1,28±0,65^b^	2,50±0,20^ab^	2,98±0,81^b^	11,11±1,75^b^	0,47±0,10^a^	0,79±0,23^a^
Steaming	1,90±0,15^a^	2,90±0,31^a^	3,22±1,13^ab^	15,12±1,89^ab^	0,46±0,09^a^	0,89±0,20^a^

Different letters in the same column indicate significant differences between treatments within each species, p < 0.05.

More specifically for salmon, canned samples (3.98mg/100g) exhibited a significant increase in Ca content compared to the control sample. On the other hand, Fe contents significantly decreased in the canned (0.61mg/100g), microwaved (1.28mg/100g), and oven-cooked (0.58mg/100g) samples compared to the control. None of the treatments produced any significant changes in Zn contents; these results are similar to those reported by Ersoy and Özeren [19] in African catfish.

Table 2 shows that Ca significantly decreased in Chilean jack mackerel samples for the different treatments of canning (11.62 mg/100g), oven cooking (12.44 mg/100g), and microwaving (11.11 mg/100g) compared to the control. For Fe, there was only a significant difference in the canned samples (2.14mg/100g), which had a lower content compared to the control. No significant changes were observed for Zn content. These results were similar to those indicated by Hosseini et al. [22] in Caspian white fish.

Different authors [25] have pointed out that seawater fish produce more Fe than freshwater fish because their food depends on the environmental conditions in which they grow and they must also cover large distances to find food, which provides more muscle exercise; Fe bioavailability is also conditioned by other nutrients such as proteins and nucleic acid [26]. Some authors have indicated that minerals are very stable during cooking whereas others have reported a higher mineral concentration after cooking because of increased dry matter [27]. Other studies have reported that mineral concentration can vary in accordance with cooking time and method [28]. Another source of variation is that minerals interact with other nutrients, such as proteins, that can alter the bioavailability of some minerals more than others [23], especially divalent minerals such as Ca, magnesium, Fe, and Zn [29], due to reduced protein quality hindering the ability to form complexes with these elements [30].

#### Fatty acid profile

The fatty acid profile for raw, canned, oven-cooked, microwaved, and steamed salmon is displayed in Table 3. The most abundant fatty acids, in descending order and expressed in % fat, were oleic acid (C18:1 n-9), palmitic acid (C16:0), linoleic acid (C18:2 n-6), DHA (C22:6 n-3), EPA (C20:5 n-3), palmitoleic acid (C16:1 n-7), stearic acid (C18:0), and myristic acid (C14:0). These results were similar to those found by Huynh and Kitts [31] who worked with capelin (*Mallotus villosus*) and Pacific herring (*Clupea harengus pallasi*).

**Table 3:** Fatty acid profile of canned, oven-baked, microwaved, and steamed salmon expressed in %.

Fatty acids	Raw	Canning	Oven cooking	Microwaving	Steaming
**C14:0**	4,43± 0,34^a^	2,79 ±0,20^b^	3,16 ±0,21^ab^	3,28± 0,13^ab^	4,97± 0,38^ab^
**C16:0**	17,88± 1,81^a^	21,30± 5,36^a^	16,77± 0,93^a^	16,74± 0,55^a^	16,75± 0,7^a^
**C18:0**	4,27± 0,28^b^	5,44 ±0,86^a^	4,37± 0,55^b^	4,28± 0,35^b^	4,36± 0,09^ab^
**ƩAGS**	**26,58± 2,23**^**a**^	**29,53± 4,67**^**a**^	**24,29 ±1,44**^**a**^	**24,31 ±0,99**^**a**^	**26,08 ±0,99**^**a**^
**C16:1n7**	5,93±0,23^a^	4,86± 0,79^b^	5,26 ±0,69^ab^	5,96 ±0,37^a^	5,18 ±0,26^ab^
**C18:1n9**	36,44±1,55^a^	34,25 ±8,87^a^	35,56 ±1,53^a^	36,68 ±1,18^a^	33,45 ±0,34^a^
**C20:1n9**	1,48± 0,61^ab^	0,78 ±0,00^b^	1,82± 0,22^a^	1,98± 0,10^a^	2,82 ±0,09^a^
**C22:1n9**	0,57± 0,13^a^	0,38± 0,00^a^	0,62± 0,03^a^	0,46± 0,27^a^	0,60 ±0,04^a^
**C24:1n9**	0,28 ±0,07^a^	ND	0,24 ± 0,02^a^	0,22 ±0,02^a^	0,24 ±0,01^a^
**ƩAGMI**	**44,7± 0,58**^**a**^	**40,28 ±9,39**^**a**^	**43,51±3,33**^**a**^	**45,29 ±0,91**^**a**^	**42,3± 0,21**^**a**^
**C18:2n6**	10,84± 0,12^a^	11,21± 2,16^a^	10,61± 0,46^a^	10,77± 0,97^a^	10,34± 0,02^a^
**C18:3n3**	2,43± 0,82^a^	1,33 ±0,01^b^	3,00± 0,08^a^	2,99± 0,21^a^	2,79± 0,02^a^
**C20:5n3**	5,83± 0,85^b^	7,43± 0,98^a^	6,17± 0,76^ab^	6,35± 0,39^ab^	7,46 ±0,14^a^
**C22:6n3**	7,64 ±0,53^bc^	10,21± 0,71^a^	8,60± 0,50^b^	7,24 ±0,68^c^	10,85 ±1,01^a^
**ƩAGPI**	**26,74± 1,87**^**a**^	**30,19 ±11,98**^**a**^	**28,39± 1,22**^**a**^	**27,35± 1,05**^**a**^	**31,44 ±0,87**^**a**^
**Ʃ n-3**	15,9± 2,00^c^	18,97 ±0,25^a^	17,78 1,11^c^	16,58± 0,84^c^	21,1 ±0,89^b^
**Ʃn-6**	10,84 ±0,12^a^	11,21 ±2,16^a^	10,61 ±0,46^a^	10,77± 0,97^a^	10,34± 0,01^a^
**n-3:n-6**	1,47± 0,20^b^	1,69 ±0,04^b^	1,68± 0,13^b^	1,54± 0,19^b^	2,04± 0,08^a^

Grouped fatty acid profile data are shown with the mean for raw (control), canned, oven-baked, microwaved, and steamed treatments.

Different lowercase letters in the same row indicate significant differences between treatments according to the LSD test p < 0.05.

ND: not determined.

The fatty acid profile for raw, canned, oven-cooked, microwaved, and steamed Chilean jack mackerel is displayed in Table 4. The most abundant fatty acids were oleic acid (C18:1 n-9), palmitic acid (C16:0), DHA (C22:6 n-3), EPA (C20:5 n-3), palmitoleic acid (C16:1 n-7), stearic acid (C18:0), and myristic acid (C14:0). These results were similar to those reported by Ferreira et al. [32] who worked with Nile tilapia (*Oreochromis niloticus*) and found that the principal fatty acids were palmitic acid (C16:0) and stearic acid (C18:0).

**Table 4:** Fatty acid profile of canned, oven-baked, microwaved, and steamed Chilean jack mackerel expressed in %.

Fatty acids	Raw	Canning	Oven cooking	Microwaving	Steaming
**C14:0**	4,20±0,4^a^	1,73 ±0,29^b^	4,35± 0,34^a^	4,14 ±0,45^a^	4,34± 0,14^a^
**C16:0**	28,41±1,23^ab^	20,98± 2,21^c^	28,56± 0,19^ab^	27,12 ±1,00^b^	30,18 ±0,92^a^
**C18:0**	8,03±0,02^ab^	5,85± 1,27^c^	7,53± 0,16^ab^	9,10 ±1,14^a^	7,16 ±0,21^bc^
**ƩAGS**	**40,64±1,63**^**a**^	**28,56± 3,25**^**b**^	**40,43± 2,07**^**a**^	**40,37± 1,60**^**a**^	**41,68 ±0,80**^**a**^
**C16:1n7**	7,01±0,09^ab^	5,21±1,29^b^	7,68± 0,51^a^	6,32± 2,04^ab^	6,63± 0,40^ab^
**C18:1n9**	33,38±0,89^a^	26,73 ±1,63^c^	30,66 ±3,32^ab^	29,06± 0,52^bc^	29,36± 0,15^bc^
**C20:1n9**	1,13±0,30^a^	1,05± 0,36^a^	1,18 ±0,31^a^	0,81 ±0,21^a^	0,92 ±0,13^a^
**C22:1n9**	0,18±0,03^a^	0,31± 0,18^a^	0,14 ±0,00^a^	0,73± 0,81^a^	0,22± 0,02^a^
**C24:1n9**	0,48±0,19^a^	0,53± 0,11^a^	0,42 ±0,01^a^	0,45 ±0,00^a^	0,41± 0,07^a^
**ƩAGMI**	**42,26±1,49**^**a**^	**33,73± 1,36**^**d**^	**40,07± 3,13**^**ab**^	**36,71 ±1,57**^**c**^	**37,54± 0,33**^**bc**^
**C18:2n6**	1,02±0,09^d^	1,64± 0,08^b^	1,95± 0,11^a^	1,21±0,03^c^	1,01 ±0,15^e^
**C18:3n3**	0,03±0,00^b^	0,63 ±0,55^a^	0,04±0,01^b^	0,01± 0,00^b^	0,04 ±0,01^b^
**C20:5n3**	5,12±0,27^c^	7,30 ±1,77^a^	6,03± 0,12^abc^	5,67±0,34^bc^	7,23±0,11^ab^
**C22:6n3**	9,19±0,54^c^	10,11± 1,32^c^	9,52 ±0,35^c^	14,27± 0,51^a^	11,07± 0,17^b^
**ƩAGPI**	**15,36±0,78**^**d**^	**19,48 ±1,07**^**b**^	**17,53± 0,57**^**c**^	**21,17 ±0,85**^**a**^	**19,35 ±0,14**^**b**^
**Ʃ n-3**	14,34± 0,80^c^	17,84± 1,08^b^	15,58± 0,46^c^	19,96± 0,82^a^	18,34± 0,23^a^
**Ʃn-6**	1,02± 0,09^d^	1,64 ±0,08^b^	1,95± 0,11^a^	1,21± 0,03^c^	1,01 ±0,15^e^
**n-3:n-6**	14,06 ±1,65^c^	10,89± 0,87^d^	7,99± 0,23^e^	16,50 ±0,44^b^	18,16± 0,38^a^

Grouped fatty acid profile data are shown with the mean for raw (control), canned, oven-baked, microwaved, and steamed treatments.

Different lowercase letters in the same row indicate significant differences between treatments according to the LSD test, p < 0.05.

Results displayed in Tables 3 and 4 for salmon and Chilean jack mackerel, respectively, were similar with the exception of linoleic acid (C18:2 n-6). These results are similar to those found by Rincon et al. [33] for linoleic acid contents in wild blackspot seabream (*Pagellus bogaraveo*) (1.28% fat) and farmed blackspot seabream (12.77% fat). Differences can be attributable to the contribution of vegetable oils in salmon feeding rather than a problem of C18:2 n-6 instability in Chilean jack mackerel.

Regarding PUFAs, no significant differences were found in the heat-treated salmon samples compared to the control, but a significant increase occurred in Chilean jack mackerel for all the treatments compared with the control. Research related to the study of PUFAs has caused controversy because some authors have reported that PUFAs in aquatic species tend to decrease during storage and cooking [7]. Other authors [9, 10, 23] have indicated that the most common culinary treatments (boiling, frying, etc.) would not have the same effect on PUFA content in many fish species, including Salmoniformes.

Omega-3 is among the PUFAs and is one of the most important groups because of its recognized benefits for human health. Higher concentrations of omega-3 found in salmon and Chilean jack mackerel samples were DHA (C22:6 n-3), EPA (C20:5 n-3), and α-linolenic acid (C18:3 n-3); these results were similar to those indicated by Hosseini et al. [22] who worked with roach (*Rutilus rutilus*). However, omega-3 fatty acids exhibited significant changes in some treatments; a significant increase was observed in canned (18.97%) and steamed (21.1%) salmon samples compared to the controls. For Chilean jack mackerel, a significant increase was detected in the canned (17.84%), microwaved (19.96%), and steamed (18.34%) samples compared to the controls. Cooking times and temperatures are important factors that could affect fatty acid content because fatty acid double bonds are more susceptible to oxidation [23].

Linoleic acid (C18:2 n-6), was the omega-6 fatty acid with the highest concentration because fatty acids with concentrations less than 0.1% were rejected. No significant differences were found in salmon treatments compared to the controls; these results coincide with those expressed by other authors [5, 22]. Heat treatments influenced omega-6 content (Table 4) in Chilean jack mackerel because a significant increase was noted in canned (1.64%), oven-cooked (1.95%), and microwaved (1.21%) samples; however, steamed samples (1.01%) significantly reduced omega-6 content compared to the control. Given that these fatty acids are susceptible to oxidation [23], this reaction is reinforced by high cooking temperatures and times [34]; for this reason, they can be affected by cooking treatments.

It is highly recommended to consume omega-3 and omega-6, but their intake must be balanced to take advantage of the health benefits they provide; an excess of either one can affect the catabolism of the other, thus reducing their incorporation in the tissues and altering their biological effects [35]. The omega-3:omega-6 relationship is known for its importance in the diet because it is a key factor for balanced eicosanoid synthesis in the organism [3]. According to the FAO/WHO recommendations, the omega-3:omega-6 relationship in the total daily diet must be greater than 1:5 (0.2) [36]. The omega-3:omega-6 relationship in salmon (Table 3) significantly increased in the steamed samples compared to the control. In Chilean jack mackerel (Table 4), this relationship significantly increased in the microwaved (16.50) and steamed (18.16) treatments, but significantly decreased for canned (10.89) and oven-cooked (7.99) samples compared to the controls. Hosseini et al. [22] indicated that cooking methods have no significant effect on the omega-3:omega-6 relationship. However, Gladyshev et al. [22] reported that the heat treatment had no significant effect on EPA, DHA, and EPA + DHA contents while the type of treatment significantly affected the omega-3:omega-6 relationship, which was reduced in all the treatments compared to the control, especially during frying and oven cooking. Although a decrease in the value of this relationship was noted in canned and oven-cooked Chilean jack mackerel, it is emphasized that these values exceeded the FAO/WHO recommendations in all treatments and in both studied species.

The EPA + DHA is the most important index within the lipid quality parameters; according to the American Heart Association, a daily intake of approximately 10 g/d EPA + DHA reduces the risk of death from coronary heart disease [22,31]. However, EPA and DHA intake recommendations are still not completely established because varied quantities have been estimated in different countries [37]; the FAO/WHO recommends daily intake of at least 500 mg/d EPA + DHA for adults [35].

To cover the nutritional needs of EPA + DHA (Recommended daily intake, RDI), it is necessary to consume 25 g of steamed salmon. However, in the case of oven-cooked salmon, the most aggressive treatment (which produces greater EPA + DHA loss), the RDI is covered by consuming 41 g. The RDI for EPA and DHA in Chilean jack mackerel for the least aggressive treatment (steaming) is covered by consuming 48 g; on the contrary, for the most aggressive treatment (microwaving), it is necessary to consume 77 g to cover the daily intake recommended by the FAO/WHO. Therefore, the daily intake recommended by the FAO/WHO is covered by small quantities of fish; these results were similar to those reported by Hosseini et al. [22].

### Conclusions

The species, time, temperature, and cooking method can be determining factors to maintain the nutritional quality of fish. Although significant changes were observed in the proximate composition, the nutritional quality of cooked fillets was not affected compared to the control. Mineral content also remained relatively stable in the different treatments in the two species under study. In general, no drastic changes were noted in the fatty acid profile during the cooking process for which, independently of the type of process, it is sufficient to consume small portions (50 to 75 g) of either salmon or Chilean jack mackerel to cover the daily intake recommended by the FAO/WHO for omega-3 fatty acids.
